# Retinal Thickness Asymmetry in Highly Myopic Eyes with Early Stage of Normal-Tension Glaucoma

**DOI:** 10.1155/2021/6660631

**Published:** 2021-01-28

**Authors:** Pei-Wen Lin, Hsueh-Wen Chang, Yi-Chieh Poon

**Affiliations:** ^1^Department of Ophthalmology, Kaohsiung Chang Gung Memorial Hospital and Chang Gung University College of Medicine, Kaohsiung, Taiwan; ^2^Department of Biological Sciences, National Sun Yat-Sen University, Kaohsiung, Taiwan

## Abstract

**Purpose:**

To investigate the retinal thickness asymmetry parameters of circumpapillary retinal nerve fiber layer (cpRNFL) and macular layers measured by spectral-domain optical coherence tomography in highly myopic (HM) patients with an early stage of normal-tension glaucoma (NTG).

**Methods:**

This cross-sectional study included 55 eyes of HM patients with early NTG and 37 eyes of HM normal participants. High myopia was defined as spherical equivalence more myopic than −6 diopters. Thickness differences and asymmetry indices (AIs) of cpRNFL between superior and inferior corresponding parts and thickness differences and AIs of the total macular layer (TML) and inner macular layers between superior and inferior hemispheres were calculated. The areas under the receiver operating characteristic curves (AROCs) were analyzed and compared.

**Results:**

In the cpRNFL asymmetry analysis, the thickness differences and AIs of cpRNFL between temporal-superior and temporal-inferior sectors (*P* < 0.0001 and *P* < 0.0001, respectively) and between superior and inferior quadrants (*P* = 0.002 and *P* < 0.0001, respectively) were significantly different between HM control subjects and HM NTG patients. In the macular asymmetry analysis, the thickness difference and AI of TML were significantly different between superior and inferior hemispheres (*P* < 0.0001 and *P* < 0.0001, respectively). The thickness difference and AI of the macular ganglion cell layer (mGCL) were significantly different between superior and inferior hemispheres (*P* < 0.0001 and *P* < 0.0001, respectively). The AROCs for thickness difference of TML (0.845) and thickness difference of mGCL (0.773) were comparable to AROCs for average cpRNFL thickness (0.842), macular retinal nerve fiber layer thickness (mRNFL) thickness (0.871), and mGCL thickness (0.822).

**Conclusion:**

In our study, HM NTG patients had retinal thickness asymmetry in cpRNFL, TML, and mGCL. The diagnostic capabilities for thickness asymmetry of TML and mGCL were comparable to the diagnostic capabilities for cpRNFL thickness, mRNFL thickness, and mGCL thickness. Asymmetry analysis of retinal thickness can be an adjunctive tool for the early detection of HM NTG.

## 1. Introduction

Glaucoma is the second leading cause of blindness worldwide [1]. It is characterized by progressive loss of retinal ganglion cells (RGC), decreased thickness of retinal nerve fiber layer (RNFL), increased cupping of the optic disc, thinning of the neuroretinal rim with notching of optic nerve head (ONH), and specific visual field (VF) defects. The retinal ganglion somas, axons, and dendrites are usually damaged in glaucomatous optic neuropathy. Optical coherence tomography (OCT) scan is commonly used for investigating the structural loss of glaucoma by measuring the thickness of the circumpapillary retinal nerve fiber layer (cpRNFL) and macular layers. However, the distribution of thickness parameters for cpRNFL is usually overlapped between normal and glaucomatous eyes. Yamada et al. reported an overlap of 68 of 92 eyes (73.9%) in the thickness of the cpRNFL between normal and glaucomatous eyes [2]. Thus, it is not easy to detect early glaucoma only by measuring the thickness changes of the cpRNFL.

In normal eyes, the distribution of RGC and the retinal thickness profiles are symmetric between the upper and lower retinal hemispheres [3, 4]. Glaucoma is usually an asymmetric disease. Mikelberg et al. reviewed the pattern of VF progression in glaucoma eyes and found that 70% of eyes had initial damage limited to a single hemifield [5]. Eyes with primary open-angle glaucoma (POAG) and primary angle-closure glaucoma had more VF damage in superior hemifield than inferior hemifield, and the differences increased with worsening disease severity [6–8]. The RNFL defects often occur before the VF defects, so the asymmetric changes of retinal thickness can occur in the early stage of glaucoma [9–15].

Myopia is an independent risk factor for glaucoma. Moderately to highly myopic (HM) eyes have a higher risk than low myopic or hyperopic eyes for the development of glaucoma [16–18]. There is a higher prevalence of normal-tension glaucoma (NTG) in myopic eyes in Asian populations [19, 20], so early detection of glaucomatous changes in myopic eyes becomes important to provide timely treatments and delay the further deterioration of visual function. However, it is often a challenge to diagnose glaucoma by the structural changes of ONH and cpRNFL in myopic eyes. The myopic eye usually has optic disc tilt and torsion, ONH deformity, shallow optic disc cupping, and peripapillary atrophy (PPA) [21, 22]. The superotemporal and inferotemporal retinal nerve fiber bundles converge temporally in myopic eyes, and it is associated with abnormal measurements of cpRNFL [23]. Because 50% of the RGC is located within the macula [24] and the RNFL is the thickest at the peripapillary retina, combined measurements of the structural changes and structural asymmetry in cpRNFL and the macular area may be helpful in diagnosing early glaucoma in myopic eyes.

Regarding the structural changes and structural asymmetry in the macular area, the Posterior Pole Asymmetry Analysis (PPAA) scan (Heidelberg Engineering, Heidelberg, Germany) can measure thickness asymmetry of macula layers between retinal hemispheres, and the diagnostic capability of macular thickness asymmetry was similar to that of cpRNFL thickness [9, 11]. In this study, we aim to investigate the thickness differences and asymmetry indices (AIs) of cpRNFL thickness parameters between superior and inferior corresponding parts and macular thickness parameters between superior and inferior hemispheres in HM patients with early stage of NTG. Areas under the receiver operating characteristic curves (AROCs) were calculated and compared to summarize the diagnostic capabilities for thickness asymmetry of cpRNFL and macular parameters for detecting the early stage of HM NTG.

## 2. Patients and Methods

### 2.1. Study Design

This cross-sectional study investigated patients with HM NTG who attended Kaohsiung Chang Gung Memorial Hospital for glaucoma medication treatment and received regular follow-up. The control subjects were from hospital staff and patients coming for ophthalmic examination. All the control subjects had no ocular disease and had not received laser procedures or ocular surgeries. High myopia was defined as spherical equivalence (SE) less than −6 diopters (D). NTG was defined as intraocular pressure (IOP) less than 21 mmHg on more than two occasions without medication, open anterior chamber angle, a glaucomatous optic disc, and cpRNFL defects with corresponding VF defects.

### 2.2. Inclusion and Exclusion Criteria

Patients were included if they had a best-corrected visual acuity of 20/40 or better and a mean deviation value greater than −6 dB on standard automatic perimetry (SAP). Patients were excluded if they had corneal degeneration, old uveitis, secondary glaucoma, optic neuropathy other than glaucoma, retinal degeneration, maculopathy, axial length (AL) greater than 28.5 mm, astigmatism exceeds ±3 D, and previous ocular trauma or refractive surgery history.

All the control subjects had open angles on gonioscopy, and normal anterior segments, ONH, and macula. The IOP measurements were less than 21 mmHg, and the SAP exams were within normal limits.

### 2.3. Ophthalmological Exams

All participants underwent a comprehensive ocular examination, which included refraction, best-corrected visual acuity, Goldmann applanation tonometry, slit-lamp biomicroscopy, gonioscopy, dilated stereoscopic exam of the ONH, central corneal thickness, AL, dilated color fundus photography, SAP, and spectral-domain OCT. The refraction was expressed as SE, which was calculated as sphere plus half of the cylinder. Central corneal thickness was measured by a Non-Contact Specular Microscope (SP-3000P, TOPCON, Tokyo, Japan), and AL was measured by IOL Master (Carl Zeiss Meditec, Dublin, California, USA). A glaucomatous optic disc was defined as the excavation of the optic disc with thinning or notching of the neuroretinal rim on color fundus photographs (TRC-50EX, TOPCON, Japan). SAP was performed with Swedish Interactive Threshold Algorithm standard 30-2 Humphrey field analyzer (HFA, Carl Zeiss Meditec, Dublin, CA). VF exams were considered reliable if the fixation losses were ≤20% and false-positive and false-negative response rates were ≤15%. A glaucomatous VF defect was defined as a cluster of 3 or more significant (*P* < 5%) nonedge contiguous points with at least 1 at the *P* < 1% level on the same horizontal meridian on the pattern deviation plot, a pattern standard deviation of 95% outside the normal limits, and a glaucoma hemifield test outside the normal limits. Glaucomatous VF defects were confirmed by two reliable SAP exams.

Spectralis OCT (Heidelberg Engineering, Heidelberg, Germany) was used to obtain the optic disc and macular scans. The fast RNFL thickness protocol was used for measuring the cpRNFL thickness. It used 1024 A-scan points from a 3.45 mm circle centered on the optic disc, and the acquisition rate of the device is 40,000 A-scans per second at an axial resolution of 3.9 *μ*m. The thickness measurements of cpRNFL were divided into 4 quadrants. The superior and inferior quadrants were further divided into nasal-superior, temporal-superior, nasal-inferior, and temporal-inferior sectors. The cpRNFL thickness in four quadrants and in four sectors was generated in the analysis report. The PPAA scan was performed to measure an 8 × 8 grid (64 separate pixels) at the macular area to assess the thickness asymmetry of macular layers. The total macular layer (TML) thickness in superior and inferior hemispheres was generated automatically in the analysis report. After the TML thickness data were obtained, the macular area was segmented to get the thicknesses of the macular retinal nerve fiber layer (mRNFL), macular ganglion cell layer (mGCL), and macular inner plexiform layer (mIPL).

The thickness differences of cpRNFL between the superior and inferior quadrants, between the nasal-superior and nasal-inferior sectors, and between the temporal-superior and temporal-inferior sectors were calculated from the RNFL thickness reports. The thickness differences of TML, mRNFL, mGCL, and mIPL between the superior and inferior hemispheres were calculated from the PPAA reports. All the thickness differences were taken as absolute values. The AIs were calculated as (thickness differences between the superior and inferior retinal layers/average thickness of the superior and inferior retinal layers) x 100. Image quality better than 20 was used for this study. Each participant underwent OCT scans to measure cpRNFL thickness and macular thickness at the same visit. One eye per participant was randomly selected for analysis.

### 2.4. Statistical Analysis

The continuous data are expressed as mean ± SD values, and the differences between glaucoma patients and control subjects were compared using an independent *t*-test. A Bonferroni's method was employed to correct for multiple comparisons. The gender of the groups was expressed as numbers and compared using the Chi-square test. AROCs were used to demonstrate the capabilities of parameters to discriminate between glaucoma patients and control subjects. Significant differences between AROCs were compared by the method of DeLong [25]. The sensitivities were calculated at 80% and 95% of specificities. A *P* value of less than 0.05 was considered statistically significant. All analyses were performed using SPSS Version 21.0 (SPSS Inc., Chicago, IL, USA) except the method of DeLong, which was performed using MedCalc19.6 (MedCalc Software, Ostend, Belgium).

## 3. Results

This study included 37 HM control subjects and 55 patients with HM NTG. The demographic data is shown in Table 1. The IOP, mean deviation, and pattern standard deviation showed significant differences between HM control subjects and patients with HM NTG.

The thickness parameters of cpRNFL and macular layers for HM control subjects and patients with HM NTG are shown in Table 2. The average thickness of cpRNFL showed significant differences between HM control subjects and patients with HM NTG. The thickness parameters of macular layers also showed significant differences between HM control subjects and patients with HM NTG. In comparison with thickness difference parameters of the cpRNFL between HM control subjects and patients with HM NTG, the thickness differences and AIs were significantly different between superior and inferior quadrants and between temporal-superior and temporal-inferior sectors. Regarding the macular thickness asymmetry assessment, the thickness differences and AIs of the TML and mGCL between superior and inferior hemispheres were significantly different between HM control subjects and patients with HM NTG. The thickness differences and AIs of mRNFL and mIPL did not show significant differences between HM control subjects and patients with HM NTG.

**Figure 1:**
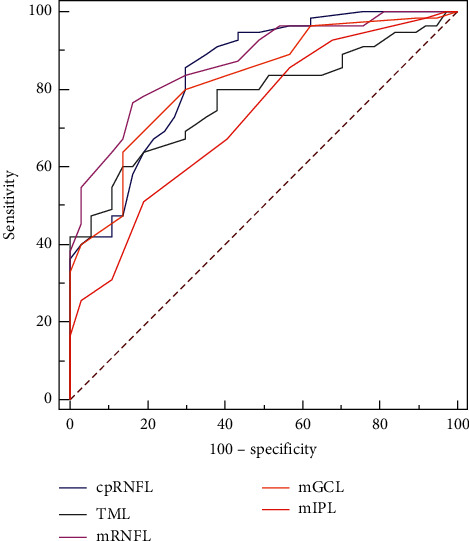
AROC curves of thicknesses of cpRNFL, TML, mRNFL, mGCL, and mIPL for discriminating highly myopic normal-tension glaucoma from highly myopic control subjects. AROC: areas under the receiver operating characteristic. cpRNFL: circumpapillary retinal nerve fiber layer thickness. TML: total macular layer thickness. mRNFL: macular retinal nerve fiber layer thickness. mGCL: macular ganglion cell layer thickness. mIPL: macular inner plexiform layer thickness.

**Figure 2:**
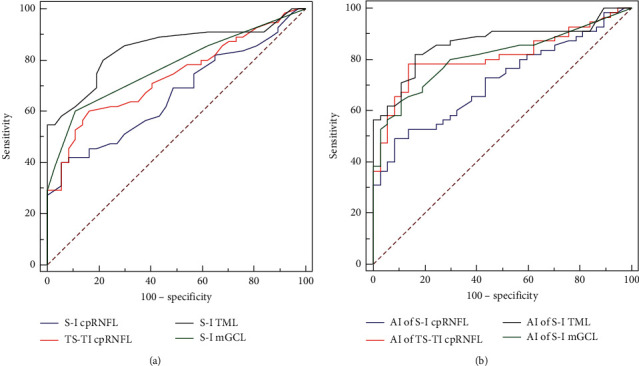
(a) AROC curves for thickness differences of S-I cpRNFL, TS-TI cpRNFL, S-I TML, and S-I mGCL for discriminating highly myopic normal-tension glaucoma from highly myopic control subjects. (b) AROC curves for AI of S-I cpRNFL, TS-TI cpRNFL, S-I TML, and S-I mGCL for discriminating highly myopic normal-tension glaucoma from highly myopic control subjects. AROC: areas under the receiver operating characteristic. AI: asymmetry index. S-I cpRNFL: thickness difference of circumpapillary retinal nerve fiber layer between superior and inferior quadrants. TS-TI cpRNFL: thickness difference of circumpapillary retinal nerve fiber layer between temporal-superior and temporal-inferior sectors. S-I TML: thickness difference of total macular layer between superior and inferior hemispheres. S-I mGCL: thickness difference of macular ganglion cell layer between superior and inferior hemispheres.

For differentiating HM NTG patients from HM control subjects, the AROCs for thickness parameters of cpRNFL and macular layers are shown in Table 3 and Figure 1. The average cpRNFL thickness and mRNFL thickness showed the highest two AROCs (0.842 and 0.871, respectively). The AROCs for thickness differences and AIs of cpRNFL and macular layers are shown in Table 3 and Figure 2. The thickness difference and AI of TML between superior and inferior hemispheres had the highest two AROCs (0.845 and 0.864, respectively). The sensitivities at 80% and 95% specificities for diagnosing HM NTG are shown in Table 3.

**Figure 3:**
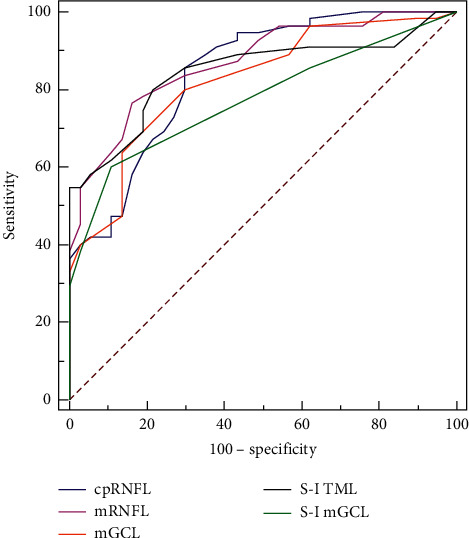
AROC curves for the thickness of cpRNFL, mRNFL, mGCL, and thickness differences of S-I TML, and S-I mGCL for discriminating highly myopic normal-tension glaucoma from highly myopic control subjects. AROC: areas under the receiver operating characteristic. cpRNFL: circumpapillary retinal nerve fiber layer thickness. mRNFL: macular retinal nerve fiber layer thickness. mGCL: macular ganglion cell layer thickness. S-I TML: thickness difference of total macular layer between superior and inferior hemispheres. S-I mGCL: thickness difference of macular ganglion cell layer between superior and inferior hemispheres.

In comparison with the diagnostic capability of each parameter, there were no significant differences in the AROCs among cpRNFL thickness, mRNFL thickness, mGCL thickness, thickness difference of TML, and thickness difference of mGCL. The diagnostic capabilities for AIs of TML and mGCL were better than the diagnostic capabilities for thickness differences of TML and mGCL. The results are shown in Table 4 and Figure 3.

## 4. Discussion

In this study, the cpRNFL thickness, TML thickness, and inner macular thickness were decreased in HM NTG. Significant thickness asymmetry of temporal sectors of cpRNFL, superior-inferior quadrants of cpRNFL, superior-inferior hemispheres of TML, and superior-inferior hemispheres of mGCL were noted in patients with HM NTG. To discriminate between HM NTG patients and HM control subjects, the thickness difference of TML and thickness difference of mGCL had comparable diagnostic capabilities to the average cpRNFL thickness, mRNFL thickness, and mGCL thickness. The diagnostic capabilities for AIs were better than the diagnostic capabilities for thickness differences.

Asymmetric changes of retinal structures and VF can be present in the early stage of glaucoma. In our previous study, we found thickness asymmetry of cpRNFL in the early stage of NTG without high myopia [15]. In this study, we also found that patients with HM NTG had thickness asymmetry of cpRNFL between temporal-superior and temporal-inferior sectors and between superior and inferior quadrants. Myopic eyes often have enlarged and oval-shaped optic discs, large areas of PPA, and tilted or torsional ONH. The torsional and tilted disc in myopic eyes can induce focal scleral tension and mechanical stress to axons at the lamina cribrosa region. The inferior tilt of the optic disc can predominantly damage the inferior vulnerable area and result in loss of the inferotemporal fibers [26]. Accordingly, asymmetric damage of RGC axons leads to thickness asymmetry of cpRNFL in HM eyes with NTG.

Macular ganglion cell bodies and their axons constitute over 30% thickness of TML. Thinning of TML in glaucoma eyes without other macular pathology can reflect the loss of inner retinal thickness [27]. In early glaucoma, small losses of macular RGC can be detected by analyzing thickness TML and mGCL. In an assessment of TML thickness asymmetry, Sullivan-Mee et al. found that macular thickness asymmetry parameters were significantly higher in the early stage of POAG than in the normal subjects [28]. We found TML thickness asymmetry in early POAG and NTG without high myopia [15]. In this study, the thickness difference and AI between superior and inferior hemispheres of TML were also significantly different between HM control subjects and HM NTG.

In an assessment of thickness asymmetry of inner macular layers, Hwang et al. evaluated the thickness differences and AIs of ganglion cell-inner plexiform layer (GCIPL) between superior and inferior hemispheres in different stages of glaucoma and found that asymmetric changes were the greatest in early glaucoma [29]. In the present study, we noted significant thickness asymmetry of mGCL between superior and inferior hemispheres. However, we did not find thickness asymmetry of mRNFL and mIPL in HM NTG. The change of RGC soma is detected before the corresponding change of RGC axon in early glaucoma [30]. More than half of RGCs are located within the macular area, and some loss of RGCs can result in thickness asymmetry of mGCL. Conversely, the mRNFL thickness and the mIPL thickness are thinner than the mGCL thickness in the macular area, and loss of the axons is later in the disease process. The thickness asymmetry of mRNFL and mIPL between superior and inferior hemispheres did not show a significant difference in early glaucoma.

Previous studies evaluated the macular thickness asymmetry in glaucoma patients, and most of them showed good diagnostic capabilities for detecting early glaucoma. Sullivan-Mee et al. compared the diagnostic capabilities of thickness asymmetry of cpRNFL and TML to discriminate between early POAG and healthy subjects. They found that the diagnostic capability for thickness asymmetry of TML was comparable to cpRNFL thickness; however, the thickness asymmetry of cpRNFL had the smallest AROC [28]. In the present study, we also found that the diagnostic capability for the thickness difference of TML was comparable to cpRNFL thickness in HM NTG eyes. The AROC for thickness difference of cpRNFL was also lower than the AROCs for cpRNFL thickness, TML thickness, and thickness difference of TML. Yamada et al. reported that the AI of mGCL had a better diagnostic capability for glaucoma than all thickness measurements and other AIs [2]. Hwang et al. investigated the glaucoma diagnostic capabilities for macular GCIPL thickness and asymmetry parameters. They found that the GCIPL thickness difference and AI of GCIPL had better glaucoma diagnostic capabilities than other GCIPL thickness parameters in differentiating early glaucoma. The AI had a better glaucoma diagnostic capability than absolute thickness differences [29]. In the present study, we found that the TML thickness difference and AI of TML had the highest two AROCs for detecting early HM NTG. The differences between our study and previous studies may be because our study included HM NTG patients whose SE was less than −6 D and the AL was 26.7 ± 0.8 mm. The refraction status and AL were different from those of Sullivan-Mee (AL: 24.4 ± 0.8 mm), Yamada (AL: 24.5 ± 1.1 mm), and Hwang (SE: −2.63 ± 3.30 D). The AL may have an influence on the measurements of macula parameters.

Macular RGC damage and cpRNFL loss are present in early glaucoma. Detecting changes of cpRNFL thickness is commonly used for the diagnosis of glaucoma. Macular GCIPL thickness has a comparable diagnostic capability to cpRNFL thickness [31]. Myopic individuals often have enlarged, tilt, or torsional optic discs and large areas of PPA that make it a challenge for early detection of HM NTG. In the present study, we noted that the TML thickness asymmetry and mGCL thickness asymmetry had comparable diagnosing capabilities to cpRNFL thickness, mRNFL thickness, and mGCL thickness. Our results suggested that in addition to detecting thickness changes in cpRNFL and macular layers, assessing thickness asymmetry in macular layers may also be helpful in diagnosing the early stage of HM NTG.

The present study has some limitations. First, our study included a small sample size, and we only evaluated the thickness asymmetry in the early stage of HM NTG. A detailed analysis of different stages of myopic NTG is needed to clarify the role of retinal asymmetry in diagnosing HM NTG. Second, the PPAA modality measured an 8 × 8 grid within the central macular area; glaucomatous damage of RGCs beyond this area could not be measured. A large scanning area for the macula will be needed to analyze retinal thickness asymmetry for identifying glaucoma. Third, the macular scan measures the central macular area; some errors may exist in myopic eyes with long AL, including the scan quality and segmentation errors. In this study, we included image quality better than 20 and AL less than 28.5 mm and excluded retinal degeneration/maculopathy to avoid poor image and unclear segmentation. Fourth, PPAA compares interhemispheric thickness differences of macula based on the line from the center of the Bruch membrane opening at disc to the fovea. Conversely, other devices draw a horizontal line passing through the fovea for interhemispheric asymmetry analysis. Different results between different OCT modalities can be present. Moreover, asymmetry analysis might have fair discriminating capabilities in moderate and advanced glaucoma patients who have symmetric structural damage. Finally, we found thickness asymmetry in cpRNFL and macula in early stage of HM NTG; there might be some extent of asymmetry in the neuroretinal rim. Further studies for asymmetry analysis of Bruch's membrane opening-based neuroretinal rim parameters may be performed to clarify the role of structural asymmetry in myopic glaucoma.

## 5. Conclusion

Thickness asymmetry of TML and mGCL was found in HM patients with an early stage of NTG. Thickness asymmetry of TML and mGCL had comparable diagnostic capabilities to thickness measurements of cpRNFL and inner macular layers. Asymmetry analysis of retinal thickness can be an adjunctive tool for the early detection of HM NTG.

## Figures and Tables

**Table 1:** Demographic data of highly myopic control subjects and highly myopic normal-tension glaucoma patients.

	Highly myopic control (*n* = 37)	Highly myopic NTG (*n* = 55)	*P* value
Age (years)	44.3 ± 7.1	45.5 ± 11.8	0.560
Male/female	16/21	34/21	0.134
CCT (um)	541 ± 33	523 ± 40	0.063
SE (diopters)	−8.22 ± 1.55	−8.60 ± 2.26	0.372
Axial length (mm)	26.4 ± 0.9	26.7 ± 0.8	0.192
IOP (mmHg)	15.4 ± 4.1	13.5 ± 3.4	0.018^*∗*^

VF			
MD (dB)	−0.93 ± 1.04	−3.20 ± 1.68	<0.0001^*∗*^
PSD (dB)	1.94 ± 0.65	4.11 ± 2.59	<0.0001^*∗*^

^*∗*^Statistically significant, NTG: normal-tension glaucoma. CCT: central corneal thickness; SE: spherical equivalence; IOP: intraocular pressure; VF: visual field; MD: mean deviation; PSD: pattern standard deviation.

**Table 2:** Average thicknesses and hemisphere thickness differences of circumpapillary retinal nerve fiber layer, total macula layer, macular retinal nerve fiber layer, macular ganglion cell layer, and macular inner plexiform layer in highly myopic control subjects and highly myopic normal-tension glaucoma patients.

	Highly myopic control (*n* = 37)	Highly myopic NTG (*n* = 55)	*P* value
cpRNFL			
Average cpRNFL thickness (um)	93.4 ± 9.6	77.8 ± 11.5	<0.0001^*∗*^
Superior-inferior cpRNFL difference (um)	13.7 ± 9.1	23.2 ± 16.2	0.002^*∗*^
AI of superior-inferior cpRNFL difference (%)	12.0 ± 8.1	25.5 ± 18.2	<0.0001^*∗*^
TS-TI cpRNFL difference (um)	19.0 ± 13.3	37.9 ± 24.5	<0.0001^*∗*^
AI of TS-TI cpRNFL difference (%)	13.7 ± 10.4	37.3 ± 23.3	<0.0001^*∗*^
NS-NI cpRNFL difference (um)	15.3 ± 12.5	19.1 ± 13.9	0.189
AI of NS-NI cpRNFL difference (%)	17.5 ± 14.3	25.2 ± 22.2	0.066

TML			
Average TML thickness (um)	289.4 ± 9.3	276.5 ± 14.1	<0.0001^*∗*^
Superior-inferior hemisphere TML difference (um)	3.9 ± 2.9	14.3 ± 9.9	<0.0001^*∗*^
AI of superior-inferior hemisphere TML difference (%)	1.3 ± 1.0	5.5 ± 4.0	<0.0001^*∗*^

mRNFL			
Average mRNFL thickness (um)	42.7 ± 4.6	33.7 ± 6.3	<0.0001^*∗*^
Superior-inferior hemisphere mRNFL difference (um)	6.8 ± 4.0	7.0 ± 5.3	0.806
AI of superior-inferior hemisphere mRNFL difference (%)	15.8 ± 8.9	21.6 ± 17.0	0.061

mGCL			
Average mGCL thickness (um)	33.0 ± 2.4	28.9 ± 3.8	<0.0001^*∗*^
Superior-inferior hemisphere mGCL difference (um)	0.8 ± 0.7	2.7 ± 2.5	<0.0001^*∗*^
AI of superior-inferior hemisphere mGCL difference (%)	2.3 ± 2.1	9.6 ± 9.8	<0.0001^*∗*^

mIPL			
Average mIPL thickness (um)	27.1 ± 2.0	25.3 ± 2.2	<0.0001^*∗*^
Superior-inferior hemisphere mIPL difference (um)	1.5 ± 1.0	2.2 ± 1.6	0.034
AI of superior-inferior hemisphere mIPL difference (%)	5.6 ± 3.9	8.7 ± 6.5	0.012

All the thickness differences were taken as absolute values. ^*∗*^Statistically significant after Bonferroni's correction. NTG: normal-tension glaucoma; cpRNFL: circumpapillary retinal nerve fiber layer; TML: total macular layer; mRNFL: macular retinal nerve fiber layer; mGCL: macular ganglion cell layer; mIPL: macular inner plexiform layer; AI: asymmetry index. TS-TI cpRNFL difference: thickness difference of circumpapillary retinal nerve fiber layer between temporal-superior and temporal-inferior sectors. NS-NI cpRNFL difference: thickness difference of circumpapillary retinal nerve fiber layer between nasal-superior and nasal-inferior sectors.

**Table 3:** Diagnostic capabilities for thickness parameters of circumpapillary retinal nerve fiber layer and macular layers, and diagnostic capabilities for hemisphere differences of circumpapillary retinal nerve fiber layer thickness parameters and macular thickness parameters for differentiating highly myopic normal-tension glaucoma from highly myopic control subjects.

Thickness parameters	Sensitivity (%) at 80% specificity	Sensitivity (%) at 95% specificity	AROC curve (95% confidence interval)
Average cpRNFL thickness	65.1	41.6	0.842 (0.761–0.923)
Average TML thickness	64.0	41.8	0.773 (0.679–0.867)
Average mRNFL thickness	78.7	57.1	0.871 (0.800–0.941)
Average mGCL thickness	70.2	41.6	0.822 (0.737–0.906)
Average mIPL thickness	51.7	27.0	0.722 (0.618–0.826)
Superior-inferior cpRNFL difference	45.8	30.6	0.663 (0.557–0.758)
AI of superior-inferior cpRNFL difference	52.7	36.4	0.714 (0.610–0.803)
TS-TI cpRNFL difference	60.9	30.8	0.734 (0.632–0.821)
AI of TS-TI cpRNFL difference	78.2	47.3	0.813 (0.718–0.886)
NS-NI cpRNFL difference	24.4	8.8	0.591 (0.484–0.693)
AI of NS-NI cpRNFL difference	27.3	12.7	0.612 (0.505–0.712)
Superior-inferior hemisphere of TML difference	76.7	57.6	0.845 (0.755–0.912)
AI of superior-inferior hemisphere of TML difference	82.6	58.2	0.864 (0.777–0.927)
Superior-inferior hemisphere of mRNFL difference	32.6	9.8	0.506 (0.400–0.612)
AI of superior-inferior hemisphere of mRNFL difference	40.0	27.3	0.567 (0.460–0.670)
Superior-inferior hemisphere of mGCL difference	64.6	44.4	0.773 (0.674–0.854)
AI of superior-inferior hemisphere of mGCL difference	70.4	54.3	0.810 (0.714–0.884)
Superior-inferior hemisphere of mIPL difference	40.1	20.1	0.601 (0.494–0.702)
AI of superior-inferior hemisphere of mIPL difference	45.5	28.6	0.656 (0.549–0.752)

AROC: areas under the receiver operating characteristics. cpRNFL: circumpapillary retinal nerve fiber layer; TML: total macular layer; mRNFL: macular retinal nerve fiber layer; mGCL: macular ganglion cell layer; mIPL: macular inner plexiform layer; AI: asymmetry index. TS-TI cpRNFL difference: thickness difference of circumpapillary retinal nerve fiber layer between temporal-superior and temporal-inferior sectors. NS-NI cpRNFL difference: thickness difference of circumpapillary retinal nerve fiber layer between nasal-superior and nasal-inferior sectors.

**Table 4:** Comparison of areas under the receiver operating characteristic curves for circumpapillary retinal nerve fiber layer thickness, macular retinal nerve fiber layer thickness, macular ganglion cell layer thickness, thickness difference of total macular layer, thickness difference of macular ganglion cell layer, asymmetry index of total macular layer, and asymmetry index of the macular ganglion cell layer.

Variable	cpRNFL	mRNFL	mGCL	S-I TML	S-I mGCL	AI of S-I TML	AI of S-I mGCL
cpRNFL	—	0.478	0.619	0.959	0.296	0.711	0.602
mRNFL	—	—	0.231	0.639	0.089	0.905	0.261
mGCL	—	—	—	0.698	0.475	0.467	0.847
S-I TML	—	—	—	—	0.150	0.011^*∗*^	0.486
S-I mGCL	—	—	—	—	—	0.073	0.010^*∗*^
AI of S-I TML	—	—	—	—	—	—	0.286
AI of S-I mGCL	—	—	—	—	—	—	—

By method of DeLong (data are *P* values). ^*∗*^Statistically significant. cpRNFL: circumpapillary retinal nerve fiber layer; mRNFL: macular retinal nerve fiber layer; mGCL: macular ganglion cell layer; AI: asymmetry index. S-I TML: thickness difference of total macular layer between superior and inferior hemispheres. S-I mGCL: thickness difference of macular ganglion cell layer between superior and inferior hemispheres.

## Data Availability

The data used to support the findings of this study are available from the corresponding author upon request.

## References

[1] Kingman S. (2004). Glaucoma is second leading cause of blindness globally. *Bulletin of the World Health Organization*.

[2] Yamada H., Hangai M., Nakano N. (2014). Asymmetry analysis of macular inner retinal layers for glaucoma diagnosis. *American Journal of Ophthalmology*.

[3] Ooto S., Hangai M., Tomidokoro A. (2011). Effects of age, sex, and axial length on the three-dimensional profile of normal macular layer structures. *Investigative Opthalmology & Visual Science*.

[4] Yamashita T., Sakamoto T., Kakiuchi N., Tanaka M., Kii Y., Nakao K. (2014). Posterior pole asymmetry analyses of retinal thickness of upper and lower sectors and their association with peak retinal nerve fiber layer thickness in healthy young eyes. *Investigative Opthalmology & Visual Science*.

[5] Mikelberg F. S., Drance S. M. (1984). The mode of progression of visual field defects in glaucoma. *American Journal of Ophthalmology*.

[6] Hart W. M., Becker B. (1982). The onset and evolution of glaucomatous visual field defects. *Ophthalmology*.

[7] Yousefi S., Sakai H., Murata H. (2018). Asymmetric patterns of visual field defect in primary open-angle and primary angle-closure glaucoma. *Investigative Opthalmology & Visual Science*.

[8] Atalay E., Nongpiur M. E., Yap S. C. (2016). Pattern of visual field loss in primary angle-closure glaucoma across different severity levels. *Ophthalmology*.

[9] Um T. W., Sung K. R., Wollstein G., Yun S.-C., Na J. H., Schuman J. S. (2012). Asymmetry in hemifield macular thickness as an early indicator of glaucomatous change. *Investigative Opthalmology & Visual Science*.

[10] Seo J. H., Kim T.-W., Weinreb R. N., Park K. H., Kim S. H., Kim D. M. (2012). Detection of localized retinal nerve fiber layer defects with posterior pole asymmetry analysis of spectral domain optical coherence tomography. *Investigative Opthalmology & Visual Science*.

[11] Kim H. S., Yang H., Lee T. H., Lee K. H. (2016). Diagnostic value of ganglion cell-inner plexiform layer thickness in glaucoma with superior or inferior visual hemifield defects. *Journal of Glaucoma*.

[12] Inuzuka H., Kawase K., Sawada A., Aoyama Y., Yamamoto T. (2013). Macular retinal thickness in glaucoma with superior or inferior visual hemifield defects. *Journal of Glaucoma*.

[13] Inuzuka H., Kawase K., Yamada H., Oie S., Kokuzawa S., Yamamoto T. (2014). Macular ganglion cell complex thickness in glaucoma with superior or inferior visual hemifield defects. *Journal of Glaucoma*.

[14] Dave P., Shah J. (2015). Diagnostic accuracy of posterior pole asymmetry analysis parameters of spectralis optical coherence tomography in detecting early unilateral glaucoma. *Indian Journal of Ophthalmology*.

[15] Lin P. W., Chang H. W., Lai I. C., Tsai J. C., Poon Y. C. (2018). Intraocular retinal thickness asymmetry in early stage of primary open angle glaucoma and normal tension glaucoma. *International Journal of Ophthalmology*.

[16] Mitchell P., Hourihan F., Sandbach J., Wang J. J. (1999). The relationship between glaucoma and myopia. *Ophthalmology*.

[17] Xu L., Wang Y., Wang S., Wang Y., Jonas J. B. (2007). High myopia and glaucoma susceptibility. *Ophthalmology*.

[18] Tham Y. C., Aung T., Fan Q. (2016). Joint effects of intraocular pressure and myopia on risk of primary open-angle glaucoma: the Singapore epidemiology of eye disease study. *Scientific Reports*.

[19] Chon B., Qiu M., Lin S. C. (2013). Myopia and glaucoma in the South Korean population. *Investigative Opthalmology & Visual Science*.

[20] Cho H.-K., Kee C. (2014). Population-based glaucoma prevalence studies in Asians. *Survey of Ophthalmology*.

[21] Park H.-Y. L., Lee K., Park C. K. (2012). Optic disc torsion direction predicts the location of glaucomatous damage in normal-tension glaucoma patients with myopia. *Ophthalmology*.

[22] Jonas J. B., Wang Y. X., Zhang Q. (2016). Parapapillary gamma zone and axial elongation-associated optic disc rotation: the Beijing eye study. *Investigative Opthalmology & Visual Science*.

[23] Leung C. K., Yu M., Weinreb R. N. (2012). Retinal nerve fiber layer imaging with spectral-domain optical coherence tomography: interpreting the RNFL maps in healthy myopic eyes. *Investigative Opthalmology & Visual Science*.

[24] Curcio C. A., Allen K. A. (1990). Topography of ganglion cells in human retina. *The Journal of Comparative Neurology*.

[25] DeLong E. R., DeLong D. M., Clarke-Pearson D. L. (1988). Comparing the areas under two or more correlated receiver operating characteristic curves: a nonparametric approach. *Biometrics*.

[26] Han J. C., Lee E. J., Kim S. H., Kee C. (2016). Visual field progression pattern associated with optic disc tilt morphology in myopic open-angle glaucoma. *American Journal of Ophthalmology*.

[27] Gupta D., Asrani S. (2016). Macular thickness analysis for glaucoma diagnosis and management. *Taiwan Journal of Ophthalmology*.

[28] Sullivan-Mee M., Ruegg C. C., Pensyl D., Halverson K., Qualls C. (2013). Diagnostic precision of retinal nerve fiber layer and macular thickness asymmetry parameters for identifying early primary open-angle glaucoma. *American Journal of Ophthalmology*.

[29] Hwang Y. H., Ahn S. I., Ko S. J. (2015). Diagnostic ability of macular ganglion cell asymmetry for glaucoma. *Clinical & Experimental Ophthalmology*.

[30] Kim Y. K., Ha A., Na K. I., Kim H. J., Jeoung J. W., Park K. H. (2017). Temporal relation between macular ganglion cell-inner plexiform layer loss and peripapillary retinal nerve fiber layer loss in glaucoma. *Ophthalmology*.

[31] Mwanza J.-C., Durbin M. K., Budenz D. L. (2012). Glaucoma diagnostic accuracy of ganglion cell-inner plexiform layer thickness: comparison with nerve fiber layer and optic nerve head. *Ophthalmology*.

